# Supplementary material for: Pathophysiological Heterogeneity of the BBSOA Neurodevelopmental Syndrome
Source: Cells. 2022 Apr 8;11(8):1260. doi: 10.3390/cells11081260 (PMC9024734; doi:10.3390/cells11081260)
Supplement: Supplementary file 1 [file cells-11-01260-s001.zip › cells-1604531-supplementary.pdf]

Supplementary Table S1. Updated list of NR2F1 variants and clinical description of patients t of BBSOAS reported patients, extended version. List of references: AK13, Al-Kateb et al. 2013; BA19, Balciuniene et al., 2019; BE18, Bertacchi et al., 2018; BE20, Bertacchi et al., 2020; BR09, Brown et al. 2009; CA09, Cardoso et al. 2009; BO14, Bosch et al. 2014; BO20, Bojanek et al., 2020; CH16, Chen et al. 2016; DI16, Dimassi et al.2016; EL17, Eldomery et al., 2017; GA21, Gazdagh et al., 2021; HF15, Hino-Fukuyo et al. 2015; HF17, Hino-Fukuyo et al. 2017; HO20, Hobbs et al., 2020; JS20, Jezela-Stanek et al., 2020; JU21, Jurkute, Bertacchi et al., 2021; KA17, Kaiwar et al. 2017; MH18, Martín-Hernández et al. 2018; MI14, Michaud et al. 2014; MI20, Mio et al., 2020; PA19, Park et al., 2019; SA13, Sanders et al., 2013; ST20, Starosta et al., 2020; RE20, Rech et al. 2020; RO20, Rochtus et al., 2020; VI17, Vissers et al., 2017; WA20 Walsh et al., 2020; ZO20, Zou et al., 2020.

Abbreviations: CB, cerebellum; CC, Corpus Callosum; CS, Corticospinal tract; D, Deletion; DBD, DNA binding domain; DD, Developmental delay; DM, Delayed myelination; DMD, Delayed motor development/poor coordination; DQ, developmental quotient; EOE/S, Early onset epilepsy/Seizures; FD, Facial dysmorphism; FI/NFI, Frameshifting indel/Non frameshifting indel; FS, Febrile seizures; GCL, ganglionic cell layer; GVI, general visual impairment; HD, Hearing defects; HP, Hippocampus; HPM, Hippocampal malrotation; ID, Intellectual disability; IQ, intelligence quotient; IS, Infantile spasms; LBD, Ligand binding domain; LV, Lateral ventricle; MCP, macrocephaly; MD, Microdeletion; MM, Missense mutation; OA, Optic atrophy; OC, optic chiasm; OCB/RB, Obsessive-compulsive/Repetitive behaviors; OD, optic disc (columns "MRI (general; optic nerve and cortical morphology)" and "Visual system defect(s) and visual deficit"), oromotor disfunction (column "Other clinical features"); ON, Optic nerve; ONH, ON hypoplasia; P/SOD, Pale/small optic disc; PDD-NOS, Pervasive Developmental Disorder-Not Otherwise Specified; PH, Bilateral Periventricular Heterotopia; RB, repetitive behavior; RNFL, retinal nerve fiber layer; TIV, Translation initiation variants; WGD, Whole gene deletion; WM, white matter.

References	LOVD Database ID; Patient ID	Age	Sex	Variant (DNA)	Variant type	Variant (protein)	MRI (general; optic nerve and cortical morphology)	DD	ID	Visual system defect(s) and visual deficit	EOE/S	ASD behavioral abnormalitie s	Hypotonia	Other clinical features	Severity index
**BR09**	BE18, #1	4yo	F	5q15 microdeletion	*De novo* deletion (400-500Kb microdeletions at breakpoints following paracentric inversion)	Deleted	Cranial nerve abnormalities	Yes	ND	ND	ND	ND	Yes	FD (malformed ears); strabismus; OD; HD	3
**CA09**	BE18, #2	7yo	M	5q14.3-q15 deletion	*De novo* deletion	Deleted	PH	Yes	Yes; speech delay	Coloboma	FS	ND	Yes	Exotropia; FD	6
**CA09**	BE18, #3	5yo	F	5q14.3-q15 deletion	*De novo* deletion	Deleted	PH	Yes	Yes; speech delay	ND	IS	ND	ND	Bilateral pes talus; minor FD	4
**CA09**	BE18, #4	5yo	M	5q14.3-q15 deletion	*De novo* deletion	Deleted	PH; HPM; MCP; polymicrogyria	Yes	Yes; speech delay	ND	Yes	ND	Yes	FD	5
**AK13**	BE18, #5; RE20, #27	8yo	M	5q15 microdeletion; chr5: 92717119-93298774 (GRCh37/hg19)	*De novo* deletion (582 Kb)	Deleted; (del. includes FLJ42709, FAM172A, POU5F2, and MIR2277)	OA (small OC)	Yes, DMD	No but speech delay	OA; GVI	No	ADHD	Yes	Uteropelvic obstruction; strabismus; FD; poor coordination; OD; HD	6
**SA13**	BE18, #6; RE20, #53	child	ND	NM_005654: c.1211G>A	*De novo* MM in LBD	p.Arg404His	ND	ND	ND	ND	ND	ASD	ND	ND	1
**BO14**	**LOVD: NR2F1_000001**; BO14, #2; BE18, #8; RE20, #13	12yo	M	c.344G>C	*De novo* MM in DBD	p.Ser113Arg	OA (small OD and OC)	Yes	No or ND	OA; P/SOD; CVI; GVI	ND	ND	Yes	FD	4
**BO14**	**LOVD: NR2F1_000002**; BO14, #1; BE18, #7; RE20, #14	2yo	F	c.339C>A	*De novo* MM in DBD	p.Arg115Pro	normal	No	Yes (IQ 48)	OA; P/SOD; small ON; CVI; GVI	ND	ND	ND	FD	2
**BO14**	**LOVD: NR2F1_000003**; BO14, #3; BE18, #9; RE20, #48	18yo	F	c.755T>C	*De novo* MM in LBD	p.Leu252Pro	ND	Yes	Yes (IQ 55-65)	P/SOD; CVI; GVI	ND	ND	Yes	FD	4
**BO14**	BO14, #4; BE18, #10; RE20, #28	24yo	F	5q15 microdeletion; chr5: 92845157-93679748 (GRCh37)	Deletion (0.83 Mb)	Deleted; (del. includes FAM172A, KIAA0825)	ND	No	Mild (IQ 61-74)	P/SOD; CVI; GVI	ND	ND	ND	FD	2
**BO14**	#5 in BO14; #11 in BE18; #24 in RE20	4yo	F	5q15 microdeletion; chr5: 91064110–93896378 (GRCh37)	*De novo* deletion (2.83 Mb)	Deleted; (del. includes FAM172A, KIAA0825, ANKRD31)	Normal or ND	Yes	No (IQ ND)	P/SOD; small ON; CVI; GVI	ND	ND	ND	FD	2
**BO14**	**LOVD: NR2F1_000004**; BO14, #6; CH16, #12; RE20, #12	35yo	F	c.335G>A	*De novo* MM in DBD	p.Arg112Lys	Normal or ND	Yes	Yes (IQ 52)	OA; P/SOD; mild GVI	ND	ASD; OCD	ND	FD	4
**HF15; HF17**	**LOVD: NR2F1_000018**; CH16, #14; RE20, #18	adult (23yo in RE20)	F	c.403C>T	*De novo* MM in DBD	p.Arg135Cys	Normal or ND	Yes, DMD	Yes (DQ <20); speech delay; non-verbal	Bilateral OA	West Syndrome; IS and FS; generalized tonic seizures	ASD traits	ND	Mild spastic quadriparesis; FD (bilateral epicanthal folds, thin upper lip, smooth philtrum, micrognathia, enlarged naris)	5
**DI16**	**LOVD: NR2F1_000057**; CH16, #15; RE20, #11	7yo	M	c.328_330delTTC	*De novo* in-frame deletion in DBD	p.Phe110del	CC thinning; LV asymmetry; septum pellucidum agenesis; abnormal gyration	Yes	Severe; speech delay; non-verbal	No or ND	West Syndrome; spasms at 3mo; IS; hypsarrhythmia; electroclinical spasms	ASD	Global	ND	6
**CH16**	**LOVD: NR2F1_000041**; CH16, #1; *BE18*, #16; RE20, #16	6yo	M	c.382T>C	*De novo* MM in DBD	p.Cys128Arg	CC thinning; WM reduction; leukodystrophy	Yes, DMD	Yes (IQ ND); speech delay; non-verbal	OA; GVI	Epilepsy with staring spells and generalized tonic-clonic seizures	ASD; self-injurious behavior; head-banging	Yes	FD; mild OD, mild HD	7
**MI14; CH16**	**LOVD: NR2F1_000075**; CH16, #2; *BE18*, #17; RE20, #17	4yo (7yo in RE20)	F	c.403C>A	*De novo* MM in DBD	p.Arg135Ser	CC thinning; WM reduction; ON malformation and OC bilateral hypoplasia	Yes, DMD	Yes (IQ ND); speech delay	Mild OA; ONH; CVI; GVI	IS; Occipital lobe epilepsy	ASD; head-banging	Profound, axial and appendicula r	Left eye esotropia and amblyopia; alacrima; FD; OD; unusually good long-term memory; high pain tolerance; touch sensitivity	7
**CH16; EL17**	**LOVD: NR2F1_000007**;	15yo	M	c.413G>A	*De novo* MM in DBD	p.Cys138Tyr	WM reduction	Global	Yes	OA; GVI	FS; Abnormal EEG during sleep	ASD; RB (persistent	No	Cyanosis at birth and mild jaundice; FD; OD	6

	CH16, #3; *BE18*, #18; RE20, #19											head-banging)			
**CH16**	**LOVD: NR2F1_000058;** CH16, #4; *BE18*, #19; RE20, #21	4yo (6.8yo in RE20)	F	c.425G>T	*De novo* MM in DBD	p.Arg142Leu	CC thinning at 7mo	Yes, DMD	Yes (IQ ND); speech delay; non-verbal	OA; P/SOD; small ON; CVI; GVI	IS; atonic seizures with markedly abnormal EEG	ND	Yes	Intermittent esotropia and intermittent exotropia; nystagmus; FD; unable to walk independently; mild OD; touch sensitivity	6
**CH16**	**LOVD: NR2F1_000010**; CH16, #5; *BE18* #20; RE20 #22	11yo	M	c.436T>C	*De novo* MM in DBD	p.Cys146Arg	CC thinning and septo-optic dysplasia	Yes, DMD	Yes (IQ ND); speech delay; non-verbal	OA; GVI	No	ASD traits; RB (self-stimulatory behaviors)	Yes	FD; unable to walk independently; OD	6
**CH16**	**LOVD: NR2F1_000024**; CH16, #6; *BE18*, #21; RE20, #47;	10yo	M	c.463G>A	*De novo* MM in DBD	p.Ala155Thr	Normal or ND	No	Mild speech delay (pronunciation; dysarthria)	No or ND	No	NO or ND	Yes	Mild bilateral hyperopia; right eye amblyopia and variable right esotropia; FD	2
**CH16**	**LOVD: NR2F1_000078**; CH16, #7; *BE18*, #22; RE20, #51	21yo	M	c.1103G>A	*De novo* MM in LBD	p.Gly368Asp	Normal or ND	Yes	Yes (IQ ND); speech delay	No or ND	First generalized seizure at 18yo	ASD; RB; aggressive behavior	No	Astigmatism; FD (cupped ears, a small mouth, sloping forehead)	4
**CH16**	**LOVD: NR2F1_000057**; CH16, #8; *BE18*, #23; RE20, #10	6yo	F	c.328_330delTTC	*De novo* in-frame deletion in DBD	p.Phe110del	CC thinning	Yes, DMD	Yes (IQ ND); speech delay; non-verbal	OA; ONH; CVI; GVI	IS	No or ND	Yes	FD (preauricular skin tag, low set ears and synophrys); unable to walk independently	6
**CH16**	**LOVD: NR2F1_000053**; CH16, #9; CH16, #24; RE20, #40	30yo	M	c.103_113delinCGCCGCCGC (c.103_113delinC in Rech et al.)	*De novo* frameshift truncation	p.Gly35Argfs*361	Normal or ND	Yes, DMD	Yes (IQ 55-69; verbal IQ 35-40 at 16yo); speech delay	OA; P/SOD; CVI; GVI	Few seizures at 3-4yo	ASD; RB including PDD-NOS at 22yo; ADHD	Yes	Recurrent otitis media; oculomotor disturbances; mild FD (synophrys, full eyebrows, high palate, retrognathia, prominent ear lobes); OD; HD	6
**CH16**	**LOVD: NR2F1_000055**; CH16, #10; *BE18*, #25; RE20, #41	21yo	M	c.291delC	*De novo* frameshift truncation	p.His97Hisfs*22	ND	Yes	Yes (IQ ND); speech delay	OA; ONH; GVI	No	ASD; OCB/RB; ADHD	Yes	Strabismus; FD; OD	5
**CH16**	**LOVD: NR2F1_000052**; CH16, #11; *BE18*, #26; RE20, #36	7yo	M	c.2T>G	*De novo* TIV	p?	Normal or ND	Yes, DMD	Yes (IQ ND); speech delay; non-verbal	OA; ONH; GVI	No	ASD (poor eye contact; emotionally labile; no aggressive behavior); RB (staring spells, head banging)	Yes	Spasticity, hyperextensibility; FD; OD	5
**CH16**	**LOVD: NR2F1_000052**; CH16, #12; *BE18*, #27; #37 in RE20, #37	4yo (7.4yo in RE20)	F	c.2T>G	*De novo* TIV	p?	CC thinning; ONH	Yes, mild DMD	Yes (IQ ND); speech delay; non-verbal	OA; ONH; P/SOD; CVI; GVI	Yes	ASD traits	Yes	Decreased tears productions; FD; OD; high pain tolerance	7
**CH16**	**LOVD: NR2F1_000048**; CH16, #13; *BE18*, #28; RE20, #35	3yo (5.7yo in RE20)	F	c.2T>C	*De novo* TIV	p.M1?	ND	Yes, DMD	Yes (FSIQ in the 40s); speech delay	OA; ONH; CVI; GVI	No	ASD; head banging	Substantial, central	Intermittent accommodative left esotropia and hyperopia; alacrima; FD; unusually good long-term memory; high pain tolerance	5
**CH16**	**LOVD: NR2F1_000048**; CH16, #14; *BE18*, #29; RE20, #38	12yo (14.6yo in RE20)	F	c.2T>C	*De novo* TIV	p.M1?	CC and CS thinning; pyramidal decussation agenesis; right *vs* left fiber directionality asymmetry	Yes, DMD	Yes (IQ ND); speech delay; non-verbal	OA; coloboma; ONH; GVI	Seizure at 3yo, complex partial, left parietal	OCB (shutting doors, skin picking, paper ripping, hand stereotypes); ADHD	Yes	Atrial septal defect; strabismus, esotropia and hyperopia; alacrima; FD (posteriorly rotated ears, epicanthal folds, broad nasal bridge, small palpebral fissures, retrognathia, high arch palate); OD; unusually good long-term memory; high pain tolerance	7
**CH16**	**LOVD: NR2F1_000042**; CH16, #15; *BE18*, #30; RE20, #39	43yo	M	c.2_4delTGGinsGGA	*De novo* TIV	p?	Cerebral malformations; bilateral HPM	Yes, DMD	Yes (IQ ND); speech delay; non-verbal	OA; GVI	Tonic-clonic seizures at 13 and 18yo years	OCB (paper cutting; organizes objects according to size)	Yes	Abnormal breathing and cyanosis at birth; slightly hypertonic in all four extremities; FD; OD	7
**CH16**	CH16, #16; *BE18*, #31; RE20, #29	8yo	F	WGD; chr5: 92856299–9305463 (GRCh37)	*De novo* deletion (0.2 Mb)	Deleted; (del. includes FAM172A, partial)	ND	Yes, DMD	Yes (IQ ND); speech delay	OA; P/SOD; pigmented maculae; GVI	No	ADHD	Yes	Ligamentous laxity; strabismus, hypertropia; FD;	5
**CH16**	CH16, #17; *BE18*, #32; RE20, #31	35yo (37yo in RE20)	M	WGD; deletion chr5: 92910393-93806933 (GRCh37)	Deletion (0.9 Mb)	Deleted; 9del. includes FAM172A; KIAA0825, partial)	ND	Yes	Yes (IQ ND); speech delay	OA; P/SOD; GVI	No	ASD; ADHD	No	Hyperextensible joints, clinodactyly of the fifth fingers, broad forefeet, large halluces, and short fourth and fifth toes; strabismus, hypermetropy, red-green colour blindness; grade 1 fovea hypoplasia; decreased tears production; FD (down slanting palpebral fissures); OD; unusually good long term memory; high pain tolerance; touch sensitivity	4
**CH16**	CH16, #18; *BE18*, #33; RE20, #32	2yo (4.7yo in RE20)	M	WGD (as patient 17 in Chen et al.)	Parental (son of CH16, #17), deletion (0.9 Mb)	Deleted	CC agenesis; DM of the EC and IC anterior limb; focal abnormality of the right CB	Yes, DMD	Yes (IQ ND); speech delay	OA; P/SOD; GVI	No	ASD traits; ADHD	Axial	Inspiration stridor and apnoea; hypo and bradykinesia; mild dystonia; laxity of hands, clinodactyly of the fifth fingers, large halluces and prominent veins on chest; decreased tears production; FD (deep set eyes, up slanting palpebral fissures, broad mouth, folded pinched helices, protruding ears, large earlobes); OD; unusually good long term memory; high pain tolerance; touch sensitivity	6

**CH16**	CH16, #19; *BE18*, #34; RE20, #30	37yo	M	WGD; chr5: 92878375-94046216 (GRCh37)	Deletion (1.2 Mb)	Deleted; (del. Includes FAM172A, KIAA0825, ANKRD31)	ND	Yes	Yes (verbal IQ 96; non-verbal IQ 70)	OA; GVI	No	ASD; PDD-NOS	No	FD	4
**CH16**	CH16, #20; *BE18*, #35; RE20, #23	6yo	F	WGD; chr5: 90566268–95580992 (GRCh37)	Deletion (5.0 Mb)	Deleted; (del. Includes FAM172A, KIAA0825, ANKRD32, MCTP30)	MCP	Yes	Yes (IQ ND)	No or ND	No	ND	Low muscle tone, normal mass and strength	Myopia; FD; OD; unusually good long-term memory	4
**KA17**	**LOVD: NR2F1_000039**; *BE18*, #36; RE20, #1	14yo (22yo in RE20)	M	c.257G>T	*De novo* MM in DBD	p.Cys86Phe	CC thinning; WM reduction; MCP	Yes, DMD	Yes (DQ<25 at 14yo); speech delay; non-verbal	OA; mild bilateral ONH; CVI; GVI	One episode of IS; left occipital onset seizure in EEG; FS	Severe ASD; RB (self-stimulating, self-injurious behavior); limited social interaction	Yes	Low serotonin and dopamine metabolites levels in CSF, delayed bone age; alacrima; FD (mild dolichocephaly, mild mid-face hypoplasia, deep set eyes with short palpebral fissures, large protruding ears with simplified helix and deficient lobuli, bilateral skin tags on the posterior aspect of the ear lobes, and micro- and retrognathia with large appearing teeth); motor apraxia, unable to walk independently; mild OD; high pain tolerance; sleep difficulties; touch sensitivity	7
**KA17**	**LOVD: NR2F1_000079**; *BE18*, #37; RE20, #52	5yo (6.7yo in RE20)	F	c.1115T>C	*De novo* MM in LBD	p.Leu372Pro	ND	Yes, DMD	Yes (IQ ND); speech delay	OA; GVI	ND	RB; ADHD	Yes	Bicuspid aortic valve and mildly dilated aortic root; anisometric amblyopia; alacrima; mild FD; slight hypertelorism, prominent synophrys, simplified cupped ear helices, and large head); mild OD; sleep difficulties	5
**VI17**	**LOVD: NR2F1_000017**	6yo	F	c.314G>A	MM in DBD	p.Gly105Asp	Cerebral malformations	ND	Yes (IQ ND)	No or ND	ND	ND	ND	ND	2
**MH18**	**LOVD: NR2F1_000040**; *BE18*, #38; RE20, #5	17yo	F	g.92921015A>G, c.286A>G	*De novo* MM in DBD	p.Lys96Glu	CC thinning	Yes	Yes (IQ ca. 30–50); speech delay	Mild OA; CVI; GVI	No	ND	Yes	Stroke-like episodes (right hemiparesis during one episode, no EEG and MRI alterations); mitochondrial complex IV deficit; elevated serum creatine kinase; convergent strabismus; behavioral arrest episodes; OD	5
**PA19**	**LOVD: NR2F1_000038**; RE20, #45; JU21, #10	8yo	M	c.513G>C	Truncation	p.Tyr171*	CC thinning	Yes	Yes; mild (IQ ca. 77–80)	OA; GVI	No	Behavioral disorders; ADHD	ND	Emmetropia; ON RNFL thinning; macula RNFL, GCL thinning; mild FD (retrognathia and external ear protrusion); poor balance and coordination	5
**BO20**	**LOVD: NR2F1_000037**; RE20, #43	23yo	M	g.92920811C>T, c.82C>T	*De novo* truncation	p.Gln28*	ONH	No	No (verbal IQ 141; nonverbal IQ 63)	OA; ONH; CVI; mild GVI	No; EEG showed rare isolated sharp waves from central regions	ASD (hand flapping and toe walking at 24mo); behavioral disorders; ADHD	Yes	Hyperbilirubinemia at birth; joint hyperextensibility; moderate to severe impairments of visual memory; bilateral RNFL thinning; mild DF (prominent ears; micrognathia; bilateral ear cupping; midface hypoplasia; flat nasal tip; prominent lips); reduced precision	5
**BE20**	**LOVD: NR2F1_000059**; BE20, #1	1yo	F	c.425G>A	*De novo* MM in DBD	p.Arg142His	CC thinning; OA (OC and nerve thinning); abnormal gyration	Yes	Yes	OA; amblyopia	IS at 8mo	ASD and ADHD traits	Yes	ND	7
**BE20**	**LOVD: NR2F1_000060**; BE20, #2	4yo	M	c.729_730delinsCT	*De novo* truncation	p.Gln244*	CC thinning; ventricular asymmetry and enlargement; abnormal gyration; polymicrogyria	Yes	Yes	No or ND	ND	Behavioral disorders	Yes	Bilateral inguinal hernias; strabismus; hypermetropia; FD (long philtrum, thin upper lip); fine motor and praxis disorders	5
**BE20**	**LOVD: NR2F1_000054**; BE20, #3; JU21, #4	9yo (6yo at MRI)	F	c.115G>T	*De novo* truncation	p.Glu39*	CC and OC thinning; CB malformation; ectopic nodular heterotopy; abnormal gyration	Yes	Yes (speech difficulties)	Severe bilateral OA; LVA	3-4 ES/y	Stereotypical movements; RB; ADHD	Yes	Precocious puberty; strabismus; moderate/high hyperopia; astigmatism; ON RNFL thinning; macula RNFL; GCL thinning	7
**BE20**	**LOVD: NR2F1_000048**; BE20, #4	3yo	F	c.2T>C	*De novo* TIV	p.?	CC thinning; cortical malformation; abnormal gyration	Yes	Yes (speech difficulties)	OA	ND	ASD and ADHD traits; behavioral disorders	No	ND	5
**BE20**	**LOVD: NR2F1_000056**; BE20, #5	6yo	F	c.292T>C	*De novo* MM in DBD	p.Tyr98His	CC thinning; OC hypoplasia; abnormal gyration	Yes	Yes	OA	ND	ASD traits; behavioral disorders; stereotypical movements	Yes	Strabismus, hypermetropia	6
**BE20**	**LOVD: NR2F1_000061**; BE20, #6	12yo	M	c.967_968delAA	*De novo* frameshift truncation	p.Lys323Serfs*73	Short CC; ON and chiasm thinning; hypoplastic olfactory lobes; abnormal gyration	Yes, DMD	Yes (speech difficulties)	OA; LVA	ND	ASD traits	No	Pectus excavatum, pes planus, scoliosis, high palate, gastroesophageal reflux, severe apnoea, strabismus, hypermetropia, tubular vision; FD (triangular face, thin upper lip, anteverted nares)	5
**ZO20**	**LOVD: NR2F1_000085**	6yo	F	c.602C>A	*De novo* truncation	p.Ser201*	ND	Mild/moderate	Mild/moderate	Bilateral P/SOD; LVA	ND	ND	ND	Colour vision impairment; bilateral RNFL and GCC thinning; FD (deep-set ears and a tall forehead)	2
**HO20**	**LOVD: NR2F1_000084**; RE20, #44	32yo	M	c.253G>T	*De novo* truncation	p.Glu85*	Normal or ND	Yes	Yes (IQ 69)	OA; GVI	Spells of behavioral arrest and non-responsiveness	ASD; auditory hallucinations and delusions; crying episodes	Yes	ND	6
**WA20**	**LOVD: NR2F1_000051**		M	NM_005654.5: c.1080del	Frameshift truncation	p.Asn362fs*33	CC, ON and OC hypoplasia; mild MCP	Apparent at 8mo	Speech delay	Severe GVI	Myoclonic astatic seizures at 2½yo	ASD	ND	Phimosis; cervico-thoracic syringomyelia; strabismus; hyperopia; FD (broad forehead and low-set small ears); HD	6

**MI20**	**LOVD: NR2F1_000034**; MI20, #1	16yo		NM_005654.4: c.313G>A	*De novo* MM in DBD	p.Gly105Ser	Benign enlargement of the subarachnoid spaces (BESS)	Yes, DMD	Speech delay; non-verbal until 2yo	Bilateral OA; GVI	Myoclonic epilepsy diagnosed at 3yo	RB	ND	Mother suffered from pre-eclampsia; synophrys; ogival palate; clinodactyly of the fifth finger; epicanthus; postural instability	6
**MI20**	**LOVD: NR2F1_000034**; #2 in MI20	16yo		NM_005654.4: c.313G>A	*De novo* MM in DBD	p.Gly105Ser	LV enlargement, intraventricular arachnoid cyst	Yes, DMD	Speech delay; non-verbal until 2yo	Bilateral OA; GVI	Myoclonic epilepsy diagnosed at 4yo	RB	ND	Mother suffered from pre-eclampsia; synophrys; ogival palate; clinodactyly of the fifth finger; DMD; postural instability	6
**ST20**	**LOVD: NR2F1_000035**	31yo	F	NM_005654.5: c.319A>G	MM in DBD	p.Lys107Glu	CC; ON; OC and optic tracts atrophy; complex pituitary cyst.	Marked and global; DMD	ND	Declining visual acuity; legally blind by 10yo	1-3yo +30 episodes of FS; occasionally with myoclonus and generalized seizures	Aggressive behavior; depression; hallucinations	Yes	Ataxia; protein-losing enteropathy; hyperinsulinism without hypoglycaemia; dental abscesses causing facial cellulitis, unilateral deep venous thrombosis after general anaesthesia; menorrhagia; intermittent mild anaemia; multiple gallstones at 25yo; bilateral enophthalmos; mild malar hypoplasia; bilateral inverted nipples, bilateral fifth finger clinodactyly, bilateral pes planus, generalized hypotonia with mildly decreased strength; brisk deep tendon reflexes; ataxia; hypotonia; OD	6
**RE20**	**LOVD: NR2F1_000065**; RE20, #33	7yo	M	c.1A>G	*De novo* TIV	p.M1?	ND	No	ND	No or ND	No	ND	Yes	Mild OD; unusually good long-term memory; high pain tolerance	1
**RE20**	**LOVD: NR2F1_000048**; RE20, #34	3yo	M	c.2T>C	*De novo* TIV	p.M1?	ND	Yes, DMD	Speech delay; non-verbal	OA; small ON; CVI; GVI	FS	ASD	Yes	Alacrima; mouth stuffing; unusually good long-term memory; high pain tolerance; HD	5
**RE20**	**LOVD: NR2F1_000067**; RE20, #2	3yo	F	c.256T>C	*De novo* MM in DBD	p.Cys86Arg	CC thinning	Yes	Speech delay; non-verbal	OA; CVI; GVI	IS	ASD traits	Yes	Unable to walk independently; OD; mild HD; sleep difficulties; touch sensitivity	7
**RE20**	**LOVD: NR2F1_000068**; RE20, #3; RO20, #170	4yo	F	c.262G>A	*De novo* MM in DBD	p.Val88Met	Normal or ND	Yes	Speech delay; non-verbal	OA; CVI; GVI	Onset at 9wo; IS; focal and partial seizures; myoclonic jerks	ASD; RB (head banging)	Yes	Decreased tears productions; unable to walk independently; OD; high pain tolerance; sleep difficulties; touch sensitivity	6
**RE20**	**LOVD: NR2F1_000009**; RE20, #4	5yo	F	c.284G>T	MM in DBD	p.Gly95Val	ND	Yes, DMD	Yes (IQ 56); speech delay	P/SOD; small ON; CVI; GVI	IS and absence seizures	ASD traits	Yes	Alacrima; mild OD; mouth stuffing; unusually good long-term memory; high pain tolerance; mild sleep difficulties; touch sensitivity	6
**RE20**	**LOVD: NR2F1_000069**; RE20, #6	8yo	M	c.290A>C	MM in DBD	p.Hys97Pro	Slightly decreased brain volume	Yes	Yes (IQ ND); speech delay; non-verbal	OA; CVI; GVI	Myoclonic seizures	ASD (severe)	Yes	Nystagmus; decreased tears production; unable to walk independently; OD; unusually good long-term memory; HD; mild sleep difficulties; touch sensitivity	7
**RE20**	**LOVD: NR2F1_000070**; RE20, #7	4yo	M	c.293A>G	*De novo* MM in DBD	p.Tyr98Cys	Abnormal	Yes, DMD	Speech delay	P/SOD; ONH; CVI; GVI	Myoclonic; absence seizures	ASD; RB (head banging); ADHD	Yes	Nystagmus; decreased tears production; OD; mouth stuffing; unusually good long-term memory; high pain tolerance; HD; sleep difficulties; touch sensitivity	7
**RE20**	**LOVD: NR2F1_000071**; RE20, #8	2yo	F	c.311A>G	*De novo* MM in DBD	p.Glu104Gly	ND	Yes	Speech delay; non-verbal	OA; CVI; GVI	No	ASD traits	Yes	Decreased tears productions; unable to walk independently; mouth stuffing; sleep difficulties	5
**RE20**	**LOVD: NR2F1_000072**; RE20, #9	5yo	F	c.323G>T	MM in DBD	p.Ser108Ile	ON thinning and small OC	Yes	Yes (IQ ND); speech delay; non-verbal	OA; small ON; CVI; GVI	No	ASD traits	Yes	Nystagmus; alacrima; OD; mouth stuffing; unusually good long-term memory; high pain tolerance; touch sensitivity	6
**RE20**	**LOVD: NR2F1_000073**; RE20, #15	2.7yo	F	c.365G>C	MM in DBD	p.Cys122Ser	ND	Yes, DMD	Speech delay; non-verbal	OA; GVI	IS	ASD traits	Yes	Nystagmus; alacrima; OD; touch sensitivity	5
**RE20**	**LOVD: NR2F1_000074**; RE20, #42	8yo	F	c.380dupA	frameshift truncation	p.Asn127Lysfs*270	ND	Yes, DMD	Speech delay	ONH; CVI; GVI	Yes	ASD; auditory hallucinations	Yes	Nystagmus; alacrima; DMD; OD; mouth stuffing; high pain tolerance; mild HD; sleep difficulties	6
**RE20**	**LOVD: NR2F1_000076**; RE20, #20	4yo	M	c.417A>T	*De novo* MM in DBD	p.Gln139His	CC thinning; DM; ON thinning and small OC	Yes, DMD	Speech delay	OA; CVI; GVI	No	ASD	Yes	Alacrima; mouth stuffing; unusually good long-term memory; high pain tolerance; sleep difficulties; touch sensitivity	6
**RE20**	**LOVD: NR2F1_000077**; RE20, #49	7yo	F	c.931G>C	MM in LBD	p.Ala311Pro	Normal or ND	Yes	Mild (FSIQ 80 below average); speech delay	P/SOD; mild GVI	Generalized Myoclonic and absence seizures	ASD	Yes	Mouth stuffing; unusually good long-term memory; sleep difficulties	6
**RE20**	**LOVD: NR2F1_000019**; RE20, #50	12yo	M	c.954G>C	*De novo* MM in LBD	p.Glu318Asp	Abnormal	No but mild DMD	No (IQ 94; performance IQ 54)	OA; CVI; GVI	Atonic; Rolandic epilepsy	ASD	No	Nystagmus; mouth stuffing; unusually good long-term memory; high pain tolerance; mild sleep difficulties	4
**RE20**	**LOVD: NR2F1_000021**; RE20, #46	4yo	F	c.1117C>T	*De novo* truncation	p.Arg373*	CC, ON and OC thinning	Yes, DMD	Mild (DQ ca. 60-70); speech delay	P/SOD; ONH; CVI; GVI	No	ASD	Yes	Nystagmus; decreased tears production; OD; mouth stuffing; unusually good long-term memory; high pain tolerance; touch sensitivity	6
**RE20**	**LOVD: NR2F1_000036**; RE20, #54	7yo	F	c.1217T>C	*De novo* MM in LBD	p.Met406Thr	Abnormal; Small ON	Yes, DMD	Yes (IQ ND); speech delay	CVI; GVI	No	ASD	Yes	Nystagmus; OD; mouth stuffing; unusually good long-term memory; high pain tolerance; sleep difficulties	6
**RE20**	**LOVD: NR2F1_000063**; RE20, #25	16.6yo	M	chr5:92414689-94864863 (hg19/GRCh37)	Maternal, deletion (2.5 Mb)	Deleted; (del. includes FLJ42709, FAM172A, MIR2277, POU5F2, KIAA0825, MIR1974, ANKRD32, MCTP1, FAM81B, TTC37)	ND	Yes	Yes (IQ ND); speech delay	OA; CVI; GVI	Absence and tonic seizures	ASD; PDD-NOS; OCD; pacing and hitting	Yes	Nystagmus; alacrima; OD; unusually good long-term memory; high pain tolerance; sleep difficulties; touch sensitivity	5
**RE20**	**LOVD: NR2F1_000064**; RE20, #26	3.6yo	M	chr5:92594997-93569402 (hg19)	Deletion (0.97 Mb)	Deleted	ND	Yes	Speech delay	OA; small ON; CVI; GVI	No	ASD	Mild	Alacrima; mild OD; mouth stuffing; unusually good long-term memory; high pain tolerance; mild touch sensitivity	5
**JU21**	JU21, #2	19yo	F	c.51_69dup	Frameshift truncation	p.Asn24Glyfs*379	Normal or ND	Yes	Yes (IQ ND); speech difficulties; learning disability	OA; ONH; CVI; LVA	No	ND	ND	Mild myopia; astigmatism; latent nystagmus; macula RNFL thinning; mild FD; poor balance and coordination	3
**JU21**	JU21, #3	19yo	M	c.91_93dupCGC	AA duplication	p.Arg31dup	Normal or ND	Yes	Yes (IQ ND); learning disability	OA; CVI; LVA	No	ND	ND	Strabismus; mild hyperopia; latent nystagmus; ON dysfunction; ON RNFL thinning; macula RNFL and GCL thinning	3

**JU21**	**LOVD: NR2F1_000069**; JU21, #5	18mo	M	c.290 A>C	*De novo* MM in DBD	p.Hys97Pro	CC, ON and OC thinning; periventricular leukomalacia; MCP	Yes, global (delayed visual maturation)	Yes (IQ ND); learning disability	OA; LVA	One episode of FS	ASD	Moderate/severe	Preterm Birth (34w and 6d). Morgagni hernia; arterial wall aneurysm; elevated liver enzymes; respiratory distress; mild hyperopia; poor balance and coordination	7
**JU21**	JU21, #6	5yo	M	c.353 T>G	*De novo* truncation	p.Leu118*	CC mild foreshortening	Yes, global	Yes (IQ ND); learning disability	OA; LVA	No	ND	Yes	Ocular torticollis; strabismus; moderate hyperopia; latent nystagmus	5
**JU21**	JU21, #7	20yo	M	c.359 dupA	(Likely) de novo truncation	p.Tyr120*	WM abnormalities	Yes, pervasive global apraxia	Yes (IQ ND); speech delay; learning disability	OA; LVA	Myoclonic epilepsy; focal impaired awareness seizures	ASD; ADHD	ND	Recurrent otitis media; strabismus; emmetropia; poor balance and coordination	6
**BA19; JU21**	**LOVD: NR2F1_000086**; JU21, #8	4yo	F	c.366 C>G	*De novo* MM in DBD	p.Cys122Trp	WM reduction; CC thinning	Yes; global	Yes (IQ ND); learning disability	OA; CVI; LVA	IS; Myoclonic epilepsy	ASD; ADHD	Yes	Strabismus; moderate hyperopia; astigmatism; mild FD; poor balance and coordination	7
**JU21**	JU21, #9	11yo	M	c.463G>A	*De novo* MM in DBD	p.Ala155Thr	WM reduction; ON thinning	Yes	Yes (IQ ND); speech delay; learning disability	CVI; LVA	No	ASD; anxiety; limited attention span	ND	Silent gastro-oesophageal reflux in infancy; frequent ear infections; strabismus; moderate hyperopia; astigmatism; ON dysfunction; poor balance and coordination	5
**JU21**	JU21, #11	3yo	M	c.599 C>G	*De novo* MM in LBD	p.Thr200Arg	Lateral and third ventricles enlargement; MCP	Global	Yes (IQ ND); learning disability	Central, steady, maintained	No	ND	Yes	Congenital heart disease, chronic lung disease with pulmonary hypertension, bilateral foot deformities; left cryptorchidism; severe preterm birth (27w and 3d); non-identical twin has evidence of DD with preterm complications; strabismus; high hyperopia; astigmatism; FD; poor balance and coordination; OD	5
**JU21**	JU21, #12	4yo	M	c.698G>A	*De novo* truncation	p.Trp233*	CC, ON and OC thinning, WM delayed maturation, brain abnormalities	Yes	Yes (IQ ND); speech difficulties	OA; microphtalmia; small ON head; CVI	No	Limited concentration and short attention span	ND	Congenital heart disease; left lacrimal duct stenosis; recurrent ear infections; microphthalmia; increased tone left ankle; sister has craniofacial abnormalities; strabismus; mild hyperopia; astigmatism; ON dysfunction; FD(asymmetric head and alopecia)	5
**JU21**	**LOVD: NR2F1_000082**; JU21, #13	9yo	F	c.1024G>A	*De novo* MM in LBD	p.Glu342Lys	Normal CC e ON; OC atrophy and defective rotation; Normal gyration	No	No	OA; ONH; LVA	No	ND	ND	Strabismus; moderate/high hyperopia; astigmatism; nystagmus; ON dysfunction; ON RNFL thinning; macula RNFL and GCL thinning	2
**JU21**	JU21, #14	29yo	F	c.1036_1047del	(Likely) *De novo* deletion in LBD	p.Glu346_Gln349del	ND	Yes; walking delay	Yes (IQ ND); speech delay; dyslexia	ONH; CVI; LVA	No	ND	ND	Frequent ear infections; cleft palate; suspected Pierre-Robin sequenced; asthma; joint hypermobility syndrome; periventricular leukodystrophy; mild myopia; latent/gaze evoked nystagmus; possible ON/RGC dysfunction; mild macula GCL thinning; poor balance and coordination	3
**JU21**	**LOVD: NR2F1_000079**; JU21, #15	49yo	F	c.1115T>C	Familial MM in LBD	p.Leu372Pro	ND	Yes; walking delay	Yes (IQ ND); speech delay; learning disability	Small ON head; CVI; LVA	No	ND	ND	Strabismus; hyperopia; nystagmus; ON/RGC dysfunction; mild macula GCL thinning; mild FD; poor coordination	3
**JU21**	**LOVD: NR2F1_000079**; JU21, #16	26yo	F	c.1115T>C	Familial MM in LBD	p.Leu372Pro	Normal or ND	Yes; walking delay	Yes (IQ ND); speech delay;	OA; ONH; CVI; LVA	One episode of FS	ND	ND	Strabismus; hyperopia; nystagmus; ON/post-retinal dysfunction; ON RNFL thinning; macula RNFL and GCL thinning; mild FD; poor coordination	4
**JU21**	**LOVD: NR2F1_000079**; JU21, #17	20yo	M	c.1115T>C	Familial MM in LBD	p.Leu372Pro	Normal or ND	Yes; walking delay	Yes (IQ ND); speech delay;	OA; CVI; LVA	No	ND	ND	Strabismus; high hyperopia; nystagmus; ON/RGC dysfunction; ON RNFL thinning; macula RNFL and GCL thinning; mild FD	3
**JU21**	JU21, #18	41yo	F	c.1118_1123del	Familial deletion in LBD	p.Arg373_Leu374del	ND	No	ND	OA; ONH; LVA	No	ND	ND	Strabismus; mild myopia; ON/RGC dysfunction; ON RNFL thinning	1
**JU21**	JU21, #19	18yo	F	c.1118_1123del	Familial deletion in LBD	p.Arg373_Leu374del	ON atrophy	No	ND	OA; ONH; LVA	No	ND	ND	Strabismus; moderate/high myopia; nystagmus; ON dysfunction with possibility of retro-chiasmal dysfunction; ON RNFL thinning; macula RNFL and GCL thinning	2
**JU21**	JU21, #20	6yo	M	c.1183G>A	*De novo* MM in LBD	p.Gly395Ser	ON atrophy; WM loss	Yes	Yes (IQ ND); learning disability	CVI; LVA	No	ND	Generalized	Strabismus; hyperopia; evidence of macular and visual pathway dysfunction; poor balance and coordination	5
**JU21**	**LOVD: NR2F1_000083**; JU21, #21	17yo (10yo at exams)	M	c.1198G>T	*De novo* truncation	p.Glu400*	CC thinning; ON and OC atrophy; abnormal gyration	Yes	Yes (IQ ND); learning disability	OA; ONH; LVA	No	ASD; behavioral disorders	ND	Moderate hypermetropia; astigmatism; mild macula RNFL and GCL thinning; poor coordination; dyspraxia	5
**JU21**	JU21, #22	7yo	F	5q15 deletion (92914091-93513068), ~599kb deletion	*De novo* whole gene deletion (599 Kb)	deleted; (del. includes FAM172A; NR2F1-AS1, partial; KIAA0825, last exon)	CC thickening; ON atrophy; cerebral vascular system abnormalities	Yes	Yes (IQ ND); mild speech delay; learning disability	OA; LVA	No	ND	ND	Strabismus; mild hyperopia; astigmatism; latent/gaze evoked nystagmus; ON RNFL thinning	4
**JS20**	**LOVD: NR2F1_000036**	10y	M	c.1217T>C	*De novo* MM in LBD	p.Met406Thr	DM	Yes	Severe (IQ ND); speech delay; non-verbal	OA; suspected ON dysplasia; GVI	Seizures from 4mo	Short attention span	ND	Recurrent infections; narrow hands with long fingers and dorsal dimpling, narrow foot with long toe and ples planovalgus; cerebral palsy; upper and lower limbs contractures; amblyopia; nystagmus horizontalis; mild FD (high forehead, large and protruding ears, widely spaced teeth); gait imbalance, broad-based gait, cerebral palsy; abnormal pain sensation	6
**GA21**	**LOVD: NR2F1_000048**	20yo	F	c.2T>C; maternally inherited Xq13.3 duplication (size: 187 kb) including NEXMIF, ABCB7, UPRT, ZDHHC15, MAGEE2, PBDC1, and MAGEE1. No maternal phenotype).	*De novo* TIV	p.M1?	Brain abnormalities; ON; OC and optic tract hypoplasia	Yes	Severe (IQ ND)	Right iris and chorioretinal coloboma; small ON; bilateral P/SOD; LVA	EEG at 12yo showed possible occipital seizures	ND	Yes	Myoclonic jerks at the beginning of sleep; Sub satisfactory growth hormone rise; height below her mid-parental range; 2-3 toe syndactyly; thick lips; long thin fingers and hands; nystagmus; poor coordination; OD	6
